# Despite Its High Salt Content, *Gochujang* Attenuates Obesity‐Induced Inflammatory Kidney Damage in Association With Gut Microbiota Modulation

**DOI:** 10.1002/fsn3.72317

**Published:** 2026-09-02

**Authors:** So‐Min Oh, Un‐Seong Na, Do‐Youn Jeong, Hayoung Woo, Anna Han

**Affiliations:** ^1^ Department of Food Science and Human Nutrition Jeonbuk National University Jeonju Jeollabuk‐do Republic of Korea; ^2^ Microbial Institute for Fermentation Industry Sunchang Korea; ^3^ Department of Nutritional Sciences University of Connecticut Storrs Connecticut USA; ^4^ K‐Food Research Center Jeonbuk National University Jeonju Jeollabuk‐do Republic of Korea

**Keywords:** *Gochujang*, gut microbiota, inflammation, kidney injury, obesity

## Abstract

Obesity causes chronic kidney damage. This study investigated the renoprotective effects of *Gochujang*, a Korean traditional fermented food, despite its high salt content. *Gochujang* extract (GE) significantly inhibited lipid accumulation and reduced inflammation‐related proteins, including TNF‐α, in renal tubular epithelial (HK‐2) cells. Additionally, *Gochujang* markedly attenuated kidney injury‐associated changes by lowering blood urea nitrogen (BUN) and kidney injury molecule‐1 (KIM‐1) levels, and by improving histopathology in high‐fat diet (HD)‐induced obese mice. Moreover, *Gochujang* altered HD‐induced gut microbiota changes by increasing diversity and reducing the *Firmicutes*‐to‐*Bacteroidetes* ratio, whereas table salt had no effect. Importantly, alterations in the gut microbiota were strongly correlated with improvements in renal injury and inflammatory indicators, implying an association between the renoprotective effects of *Gochujang* and gut microbiota modulation. These findings highlight the potential of *Gochujang* to attenuate obesity‐related kidney injury‐related changes despite its high salt content.

## Graphical abstract

*Gochujang,* a traditional Korean fermented food, reduced lipid accumulation and inflammatory responses in HK‐2 cells and attenuated kidney injury‐related changes in high‐fat diet‐induced obese mice. These effects were accompanied by modulation of the gut microbiota, supporting the potential renoprotective effects of *Gochujang* despite its high salt content.

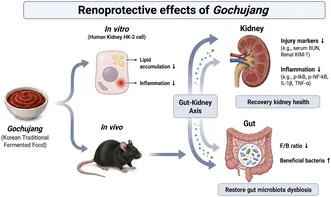

## 1 Introduction

Obesity is a major global health issue and increases the risk of kidney injury as one of its complications (Monu et al. 2022). One primary causal factor of obesity is an imbalanced diet, including high‐fat diets (HD) and high‐salt (HS) diets, which not only induce weight gain but also have significant effects on kidney health (Yu et al. 2022). Without timely and appropriate intervention, obesity‐related kidney injury progresses into chronic kidney disease (CKD) because of persistent oxidative stress and inflammatory responses (Ha et al. 2022). Recent data indicate that approximately 44% of obese individuals are diagnosed with CKD in the United States (Kreiner et al. 2023). Furthermore, CKD patients require dialysis or kidney transplantation, leading to long‐term patient management and additional health complications (B. Li et al. 2024). Therefore, developing effective dietary patterns and food‐based interventions is crucial to maintaining kidney health in obese individuals (Arabi et al. 2023).

Obesity‐induced kidney injury is characterized primarily by fat accumulation in the kidney and heightened inflammatory responses (Stasi et al. 2022). These pathological outcomes elevate blood urea nitrogen (BUN) levels and increase the expression of kidney injury biomarkers, such as kidney injury molecule‐1 (KIM‐1) (Chen et al. 2021). Recent pre‐clinical studies suggest that certain foods and/or dietary bioactive compounds mitigate diet‐induced kidney injury (González 2020). For instance, curcumin alleviates structural kidney damage and reduces renal fat accumulation, inflammation, and fibrosis by lowering blood lipid levels and renal KIM‐1 levels in obese mice induced by high‐fat or high‐fructose diets (Meléndez‐Salcido et al. 2023). Furthermore, various fermented foods have been reported to have renoprotective effects due to their anti‐inflammatory properties (Shahbazi et al. 2021). For example, fermented soybeans significantly suppress kidney disease progression by alleviating inflammation, suggesting the renal‐advantageous potentials of Korean traditional fermented foods (KTFF), such as *Doenjang* and *Gochujang* (Paul et al. 2023).

The gut microbiota plays a crucial role in host health by regulating various metabolic processes and reducing inflammation in the gastrointestinal tract (Wu et al. 2021). Importantly, diet significantly influences gut microbiota composition, leading to the development and/or improvement of multiple metabolic diseases (Mao et al. 2023). For example, Ang et al. reported that polyphenol‐rich oolong tea alleviates hepatic inflammation by improving gut microbiota imbalance in HD‐induced obese mice (A. Li et al. 2022). However, most previous studies on the impact of the gut microbiota on host health have focused primarily on diseases related to liver and adipose tissue metabolism. Recent research suggests that the gut microbiota also plays a significant role in kidney function and disease. Indeed, a recent study demonstrated that a decrease in *Lactobacillus* and *Bifidobacterium* is strongly associated with increased kidney inflammation, oxidative stress, and CKD progression (Huang et al. 2022). Thus, there is a pivotal need to investigate the impact of specific foods and/or dietary interventions on the gut‐kidney axis.

As a KTFF, *Gochujang* is a representative synbiotic food containing various beneficial microorganisms and bioactive compounds generated during fermentation (Ryu et al. 2021). Previous studies have demonstrated that *Gochujang* exhibits multiple health benefits, including anti‐inflammatory, anti‐cancer, and antioxidant effects (Edward et al. 2023; Kim et al. 2023; H. Lee, Song, et al. 2023; H. Y. Lee et al. 2024; Park et al. 2024; E.‐B. Seo et al. 2024). *Gochujang* improves constipation by modulating gut microbiota imbalance and enhancing gastrointestinal motility in loperamide‐induced constipation mice (Kim et al. 2023). However, the effects of *Gochujang* on kidney function, particularly in obesity‐induced renal injury, have not yet been fully investigated. Furthermore, the high salt content of KTFF has raised concerns regarding its potential risk for kidney injury and hypertension (Park et al. 2024). Interestingly, the consumption of *Doenjang*, another well‐known KTFF, did not increase the risk of hypertension despite its high salt content, whereas table salt significantly increased the risk of hypertension, a pattern defined as the “Korean paradox” (Mun et al. 2019). This finding suggests that table salt and salt in KTFF may have different effects on renal health.

Therefore, the aims of this study were (1) to evaluate the renoprotective effects of *Gochujang* in obesity‐induced kidney injury, (2) to elucidate its detailed underlying cellular and molecular mechanisms, and (3) to study the association between *Gochujang*‐induced gut microbiota modulation and kidney damage‐related indicators. Lastly, the salt group was included to compare the distinct effects of table salt and salt in KTFF on kidney health.

## 2 Materials and Methods

### 2.1 Preparation of *Gochujang*

The microbial composition of *Gochujang* was analyzed using Next‐Generation Sequencing (NGS) at the Microbial Institute for Fementation Industry (Sunchang, Jeollabuk‐do, Republic of Korea). Among 76 *Gochujang* samples examined, two *Gochujang* samples were selected based on their high composition of bacteria (*Bacillales*) at the order level (KTG #1 and #2).

For the *Gochujang* extract (GE), KTG #1 and #2 were combined (1:1) and processed as described by Seo et al. (E.‐B. Seo et al. 2024). Briefly, the samples were freeze‐dried, ground, and extracted twice with 80% ethanol. The combined supernatants were filtered, concentrated, and freeze‐dried to obtain powdered GE, which was stored at −70°C. For cell treatment, GE was dissolved in DMSO, diluted in PBS (final 1% DMSO), and filtered through a 0.45 μm membrane.

### 2.2 Analysis of Total Phenolic Content (TPC) and Total Flavonoid Content (TFC) of GE

TPC was determined using gallic acid (Sigma, USA, G7384) as the standard, with absorbance measured at 760 nm. TFC was quantified using rutin (Sigma, USA, R5143) as the standard, with absorbance measured at 420 nm. Absorbance was measured with a TECAN Infinite M Plex microplate reader using i‐control 2.0 software.

### 2.3 Analysis of the Radical Scavenging Activity of GE

Antioxidant activity of each GE (KTG #1 and KTG #2) was evaluated using 2,2′‐Azino‐bis (3‐ethylbenzthiazoline‐6‐sulfonic acid) (ABTS) and 2,2‐diphenyl‐1‐picrylhydrazyl (DPPH) radical scavenging assays. For the ABTS assay, ABTS (Sigma, USA, A1888) radicals were generated by mixing with potassium persulfate (Sigma, USA, 216224) and incubating for 24 h, after which they reacted with GE at concentrations of 0.125, 0.25, 0.5, 1, and 2 mg/mL. The absorbance was measured at 734 nm using a microplate spectrophotometer (TECAN, INFINITE M PLEX). For the DPPH assay, GE at concentrations of 0.125, 0.25, 0.5, 1, and 2 mg/mL was reacted with 0.2 mM DPPH solution (BIOZOA Biological Supply, Seoul, Republic of Korea, 890,132) in the dark for 30 min, and the absorbance was measured at 517 nm.

### 2.4 Cell Culture

HK‐2 cells (CRL‐2190, ATCC, USA) were routinely cultured in DMEM (HyClone, USA) supplemented with 10% fetal bovine serum (Gibco, USA) and 1% penicillin–streptomycin (100 IU/mL of penicillin and 100 μg/mL of streptomycin) (Sigma, USA) at 37°C, 95% air, and 5% CO_2_.

### 2.5 Cell Viability Assay

To assess the toxicity of GE on HK‐2 cells, the EZ‐CYTOX kit (DoGenBio, Seoul, Republic of Korea) was used, following a previously established protocol (E.‐B. Seo et al. 2024). Cells (4 × 10^3^ cells/well) were seeded in 96‐well plates (SPL, USA) and incubated for 24 h to allow adherence. After GE treatment, the medium was replaced with EZ‐CYTOX reagent mixed in culture medium (1:10), and 100 μL was added per well. Following a 1 h incubation and gentle shaking, absorbance was measured at 450 nm using a microplate reader (TECAN, INFINITE M PLEX) with i‐control 2.0 software.

### 2.6 Palmitic Acid Accumulation in HK‐2 Cells and Oil Red O Staining

Palmitic acid (PA) accumulation in HK‐2 cells was performed according to the protocol described in a previous study (S. H. Seo et al. 2019). Cells were seeded at 5 × 10^5^ cells per well in 6‐well plates and allowed to stabilize for approximately 5 days. Subsequently, cells were treated with a complex of PA (Sigma, USA, P9767) and BSA (Sigma, USA, A6003) at concentrations of 800 μM PA and 1.5% BSA for 24 h. The treatment groups were as follows: Control (1.5% BSA), PA (PA‐BSA complex), and PA + GE (PA‐BSA treatment followed by additional treatment with GE at 0.25 or 0.5 mg/mL for 24 h).

Lipid accumulation in the cells was assessed by fixing cells with 4% paraformaldehyde (PFA), followed by Oil Red O (ORO; Sigma‐Aldrich, USA, O1391) staining. Stained cells were observed under a light microscope (Thermo Fisher, EVOS M7000, AMF7000), and lipid accumulation was quantified using ImageJ software.

### 2.7 IL‐1β Treatment in HK‐2 Cells

HK‐2 cells were treated with IL‐1β (R&D Systems, USA, #201‐LB) at concentrations of 0, 1, 2, and 5 μg/mL for 6 h. For subsequent experiments, cells were either treated with growth media as a control, treated with IL‐1β (5 μg/mL) for 6 h, or pre‐treated with GE (0.25 mg/mL) for 24 h, followed by IL‐1β (5 μg/mL) treatment for 6 h.

### 2.8 Animal Studies

This study was approved by the Institutional Animal Care and Use Committee of Jeonbuk National University (JBNU 2021‐0112). Male C57BL/6 mice, 3 weeks old, were purchased from DooYeol Biotech (Seoul, Republic of Korea). The mice were housed under controlled conditions with a 12 h/12 h light/dark cycle at 23°C ± 1°C and a relative humidity of 60% ± 5%. To evaluate the effects of HD on kidney disease and the efficacy of *Gochujang*, mice were acclimatized for 3 weeks, and then randomly assigned to five groups (*n* = 7): ND (normal diet, 10% fat), HD (high‐fat diet, 60% fat), SALT (HD with 0.7% table salt), KTG #1 (HD with 10% Korean Traditional *Gochujang* #1), and KTG #2 (HD with 10% Korean Traditional *Gochujang* #2). The percentage of *Gochujang* in the KTG diets was determined based on previous studies investigating the physiological effects of KTFFs in animal models (Edward et al. 2023; E.‐J. Lee, song, et al. 2023; H. Lee, song, et al. 2023). In this study, “table salt” refers to purified NaCl added directly to the diet, whereas sodium in the KTG diets was derived from salt added to *Gochujang* during its manufacturing and fermentation. The nutritional composition of *Gochujang* and the diet formulation are shown in Figure S1A,B, respectively. Accordingly, the SALT diet, containing 0.7% table salt, was formulated to match the NaCl content of the KTG diets. In addition, for the KTG diets, the amounts of casein, maltodextrin, and lard were adjusted to compensate for the protein, carbohydrate, fat, and energy provided by 10% *Gochujang*. Mice had free access to food and water. Weekly body weight and diet intake (three times a week) were recorded, and energy efficiency was calculated. After 13 weeks of experimental diets, mice were anesthetized with Alfaxan (6.5 mg/kg body weight, intramuscular injection), and blood was collected via the retro‐orbital blood sinus. Following blood collection, the animals were euthanized by cervical dislocation, and serum and tissues were harvested for further analyses.

### 2.9 Serum Biochemical Measurements

Serum samples were centrifuged at 3000 × g for 15 min (4°C). Total cholesterol (TC) and triglyceride (TG) levels were measured using commercial kits (Asan Pharmaceutical Co, Seoul, Republic of Korea; TC kit; Cat #. AM202, TG kit; Cat #. AM157S). In addition, BUN levels were measured using a commercial kit (Thermo Fisher, USA, Cat #. EIABUN) according to the manufacturer's instructions.

### 2.10 Histological Staining

The kidney tissue was fixed in 4% PFA and processed with hematoxylin and eosin (H&E) staining at KP&T Company (Cheongju, Republic of Korea). The stained samples were analyzed through an optical microscope (Thermo Fisher, EVOS M7000, AMF7000).

### 2.11 Western Blot

Kidney tissue and HK‐2 cells were homogenized in lysis buffer containing RIPA buffer (Biosesang, Seoul, Republic of Korea), protease inhibitor, and phosphate inhibitor cocktail (EMD Millipore Corp., Burlington, MA, USA), and the supernatant was collected after centrifugation (4°C, 14,000 × g, 15 min). Proteins were electrophoresed on 8%–12% SDS polyacrylamide gels and transferred to polyvinylidene difluoride membranes (Bio‐Rad Laboratories, Hercules, CA, USA). Subsequently, blotting was performed using primary antibodies: β‐actin (Sigma, 2066, 1:3000), p‐ERK (Cell Signaling, 9101, 1:1000), p‐JNK (Cell Signaling, 9251, 1:500), p‐NF‐κB (Santa Cruz, 136,548, 1:500), TNF‐α (Santa Cruz, 52,746, 1:500), IL‐1β (Santa Cruz, 1222, 1:500), KIM‐1 (Abcam, 78,494, 1:1000), α‐SMA (Cell Signaling, 19,245, 1:1000), and p‐IκB (Santa Cruz, 8404, 1:500). Then, the membrane was washed and incubated with secondary antibodies such as anti‐rabbit or anti‐mouse.

### 2.12 Gut Microbiota Analysis

At the end of the intervention period, fresh feces were collected, stored at −80°C, and subsequently processed at the Microbial Institute for Fermentation Industry (Sunchang, Jeollabuk‐do, Republic of Korea). Microbial community composition, α‐diversity, and β‐diversity were analyzed using EzbioCloud 16S‐based Microbiome Taxonomic Profiling software (ChunLab, Seoul, Republic of Korea). Fecal microbiota analysis followed a previously published protocol (Kim et al. 2023; S.‐B. Lee et al. 2026). Briefly, DNA was isolated using the FastDNA Spin Kit (MP Biomedicals, Santa Ana, CA, USA), and the bacterial 16S rRNA V1–V3 region was amplified with primers 27F (5′‐GAGTTTGATCMTGGCTCAG‐3′) and 518R (5′‐WTTACCGCGGCTGCTGG‐3′). PCR involved 10 cycles with annealing at 60°C, followed by 20 cycles at 55°C, with denaturation at 94°C and extension at 72°C. Taxonomic classification was performed using the Ez‐Taxon database (PKSSU version 4.0). Detailed information regarding the sequencing platform, sequence‐processing pipeline, associated software versions, and sequencing depth was unavailable for this dataset.

### 2.13 Statistical Analysis

All results are expressed as mean ± standard error of the mean (SEM). Data analysis was performed using one‐way ANOVA in SPSS version 23.0 (SPSS, USA), followed by Duncan's Multiple Range Test; the statistical significance criterion was set at *p* < 0.05. When comparing two groups, a *t*‐test was used, and *p < 0.05, **p < 0.01, and ***p < 0.001 were considered statistically significant.

## 3 Results

### 3.1 Characteristics of *Gochujang*

**FIGURE 1:**
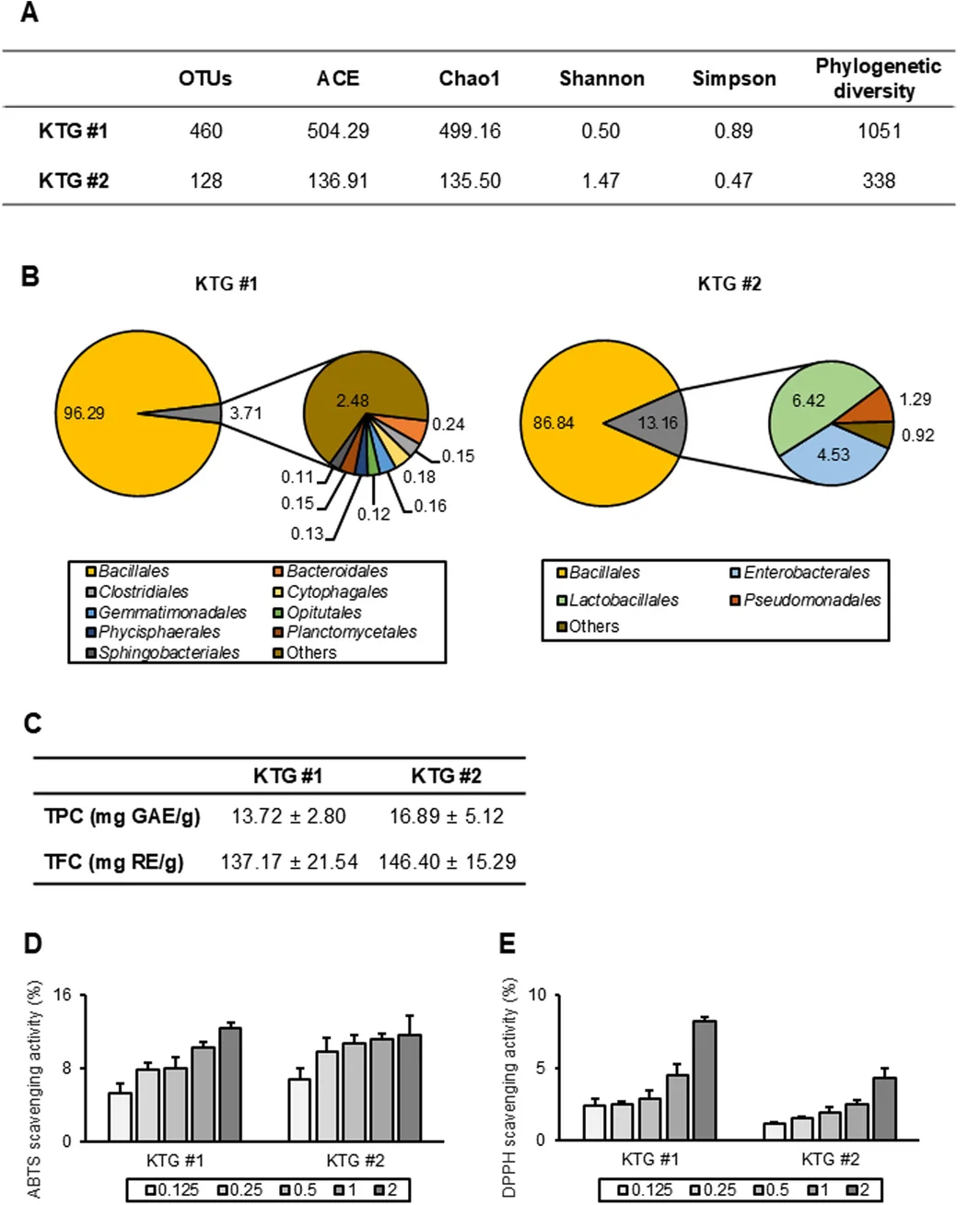
Characteristics of *Gochujang*. (A) α‐diversity of *Gochujang*. (B) Microbial composition of *Gochujang* at the order level. (C) TPC and TFC of GE at 0.5 mg/mL. (D) ABTS radical scavenging activity of GE and (E) DPPH radical scavenging activity of GE. KTG #1, HD with 10% of KTG #1 *Gochujang* group; KTG #2, HD with 10% of KTG #2 *Gochujang* group.

To compare the two types of *Gochujang*, the proportion and composition of microbial taxa were analyzed. KTG #1 showed higher OTUs, ACE, Chao1, Simpson, and phylogenetic diversity than KTG #2, whereas the Shannon index was higher in KTG #2 (Figure 1A). At the order level, the relative abundance of *Bacillales* was 96.29% in KTG #1 and 86.84% in KTG #2, respectively (Figure 1B). In addition, KTG #1 was predominantly composed of the *Bacillales*, while KTG #2 contained a relatively wider variety of bacterial orders, including *Bacillales*, *Enterobacterales*, and *Lactobacillales* groups.

The nutritional composition of each *Gochujang* is shown in Figure S1A. Specifically, 100 g of KTG #1 contained 41.52 g of carbohydrates, 5.62 g of protein, 2.83 g of fat, and 6.6 g of NaCl, while 100 g of KTG #2 contained 39.02 g of carbohydrates, 5.86 g of protein, 3.01 g of fat, and 6.8 g of NaCl. Additionally, to characterize the chemical properties of *Gochujang*, the TPC and TFC of each GE were measured at a concentration of 0.5 mg/mL (Figure 1C). KTG #2 exhibited higher TPC and TFC than KTG #1, containing 16.89 ± 5.12 mg GAE/g of TPC and 146.40 ± 15.29 mg RE/g of TFC, compared with 13.72 ± 2.80 mg GAE/g of TPC and 137.17 ± 21.54 mg RE/g of TFC in KTG #1, respectively. Furthermore, ABTS and DPPH radical‐scavenging activities were measured to assess the antioxidant capacity of each GE sample. Both ABTS and DPPH radical‐scavenging activities increased in a concentration‐dependent manner (Figure 1D,E). Taken together, these results indicate that KTG #1 and KTG #2 differed in their chemical and antioxidant profiles and in their microbial composition.

### 3.2 GE Suppresses Lipid Accumulation and Inflammatory Activation in HK‐2 Cells

**FIGURE 2:**
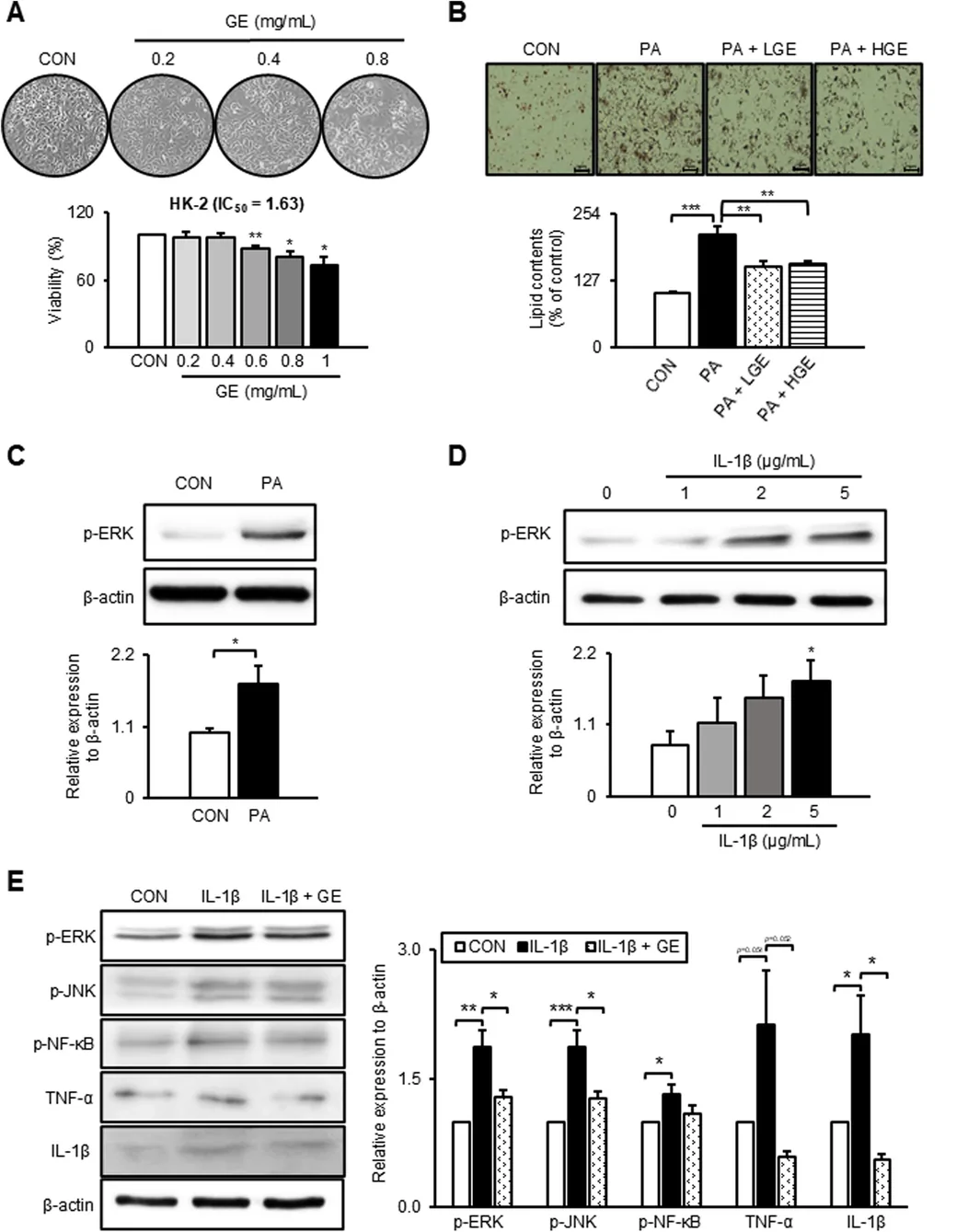
GE suppresses lipid accumulation and inflammatory activation in HK‐2 cells. (A) HK‐2 cells were treated with GE at 0.2, 0.4, 0.6, 0.8, and 1 mg/mL for 24 h. Cell viability was measured to determine the IC_50_ value. (B) Lipid accumulation was assessed by Oil Red O staining (40X, scale bar = 75 μm) following treatment with PA (800 μM, 1.5% BSA), with or without co‐treatment of low (0.25 mg/mL, LGE) or high (0.5 mg/mL, HGE) concentrations of GE. Quantification was expressed as % of the control. (C) Expression of p‐ERK in HK‐2 cells treated with PA for 24 h was evaluated by Western blot. (D) HK‐2 cells were treated with IL‐1β (0, 1, 2, or 5 μg/mL) for 6 h. (E) Western blot analysis of p‐ERK, p‐JNK, p‐NF‐κB, TNF‐α, and IL‐1β in IL‐1β‐treated HK‐2 cells with or without GE co‐treatment. β‐actin was used as a loading control. Values are mean ± SEM (*n* = 5). Comparisons involving more than two groups were performed using one‐way ANOVA followed by Duncan's multiple‐range test, whereas comparisons between two groups were performed using an unpaired *t*‐test. *p < 0.05, **p < 0.01, ***p < 0.001. ERK, extracellular signal‐regulated kinase; GE, *Gochujang* extract; HGE, high *Gochujang* extract (0.5 mg/mL); HK‐2, human renal tubular epithelial cells; IL‐1β, Interleukin‐1 beta; JNK, c‐Jun N‐terminal kinase; LGE, low *Gochujang* extract (0.25 mg/mL); NF‐κB, nuclear factor kappa B; p‐, phospho‐; PA, palmitic acid; TNF‐α, tumor necrosis factor alpha.

To determine the optimal GE concentration for HK‐2 cells, the cells were treated with concentrations ranging from 0.2 to 1 mg/mL. With GE treatment, cell viability began to decrease significantly at 0.6 mg/mL, and the IC_50_ was 1.63 mg/mL (Figure 2A). Based on this result, 0.25 mg/mL (low, LGE) and 0.5 mg/mL (high, HGE) were selected for subsequent experiments.

Next, HK‐2 cells were treated with PA to induce lipid accumulation and evaluate the lipid‐accumulation inhibitory effects of GE. PA treatment significantly increased lipid content compared to CON, while both GE groups (LGE and HGE) showed a significant reduction in lipid accumulation compared to PA treatment (Figure 2B). PA treatment significantly upregulated the expression of p‐ERK compared to CON (Figure 2C). Then, HK‐2 cells were directly treated with IL‐1β to study the inflammation‐repressive effects of GE in HK‐2 cells. p‐ERK expression was dose‐dependently increased by IL‐1β treatment (Figure 2D), and 5 μg/mL was therefore selected as the concentration for subsequent experiments. Compared with CON, IL‐1β treatment significantly elevates the levels of p‐ERK, p‐JNK, p‐NF‐κB, TNF‐α, and IL‐1β (Figure 2E). In contrast, GE pretreatment markedly lowered the expression of p‐ERK, p‐JNK, TNF‐α, and IL‐1β compared with IL‐1β treatment, whereas p‐NF‐κB was slightly downregulated.

These findings suggest that *Gochujang* may ameliorate HD‐induced kidney damage by suppressing lipid accumulation and the inflammatory response.

### 3.3 Effects of *Gochujang* on Body Weight Gain and Lipid Indicators in HD‐Induced Obese Mice

**FIGURE 3:**
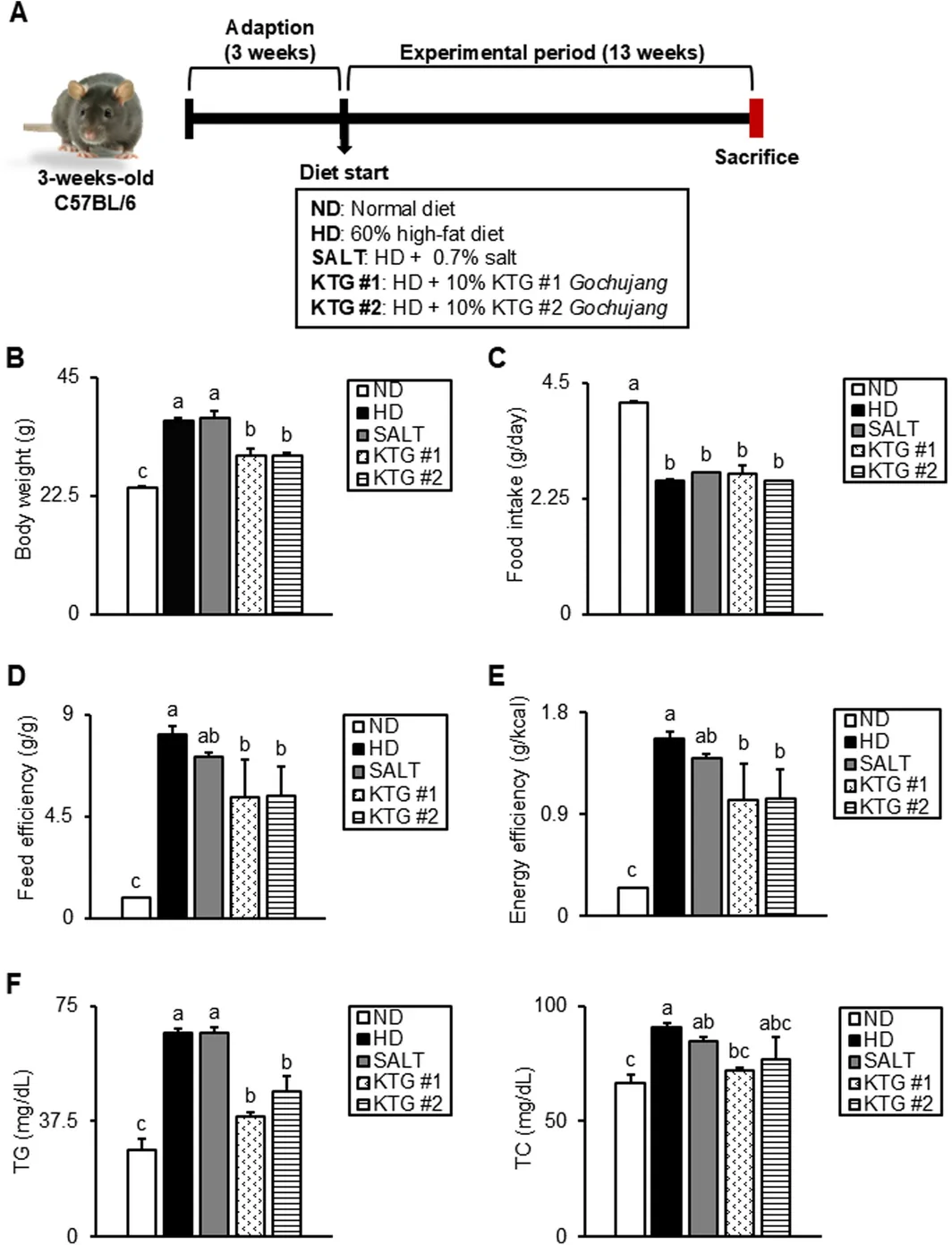
Effects of *Gochujang* on body weight gain and lipid indicators in HD‐induced obese mice. (A) Experimental period. (B) Final body weight of mice. (C) Food intake. (D) Feed efficiency. (E) Energy efficiency. (F) Serum triglyceride (TG) and total cholesterol (TC) levels. Values are mean ± SEM. (a‐c) means with the different letters (*a* > b > c). ND, normal diet group; HD, high‐fat diet group; SALT, HD with 0.7% salt group; KTG #1, HD with 10% of KTG #1 *Goc*huja*ng* group; KTG #2, HD with 10% of KTG #2 *Gochujang* group.

As GE significantly improves lipid accumulation and inflammation at the cellular level (Figure 2), the renoprotective effects of *Gochujang* were investigated in HD‐induced obese mice (Figure 3A). To confirm HD‐induced obesity and the effect of *Gochujang* on body weight (BW) gain in HD‐induced obese mice, BW on the final day of the experiment was measured. Compared to the ND group, the BW of both HD and SALT groups was significantly increased (Figure 3B). All KTG groups exhibited significantly lower BW gain compared to HD and SALT groups. Among HD groups, there was no significant difference in food intake (Figure 3C). Compared with the ND group, HD and SALT groups had a higher feed efficiency and energy efficiency than both KTG groups (Figure 3D,E). The analysis of serum lipid parameters showed increased TG and TC levels in both HD and SALT groups relative to the ND group. TG levels were significantly reduced in both KTG groups compared with HD and SALT groups. In addition, TC levels tended to decrease in KTG groups compared with the HD and SALT groups (Figure 3F).

These results indicate that *Gochujang* has anti‐obesity effects and ameliorative effects on dyslipidemia in HD‐induced obese mice.

### 3.4 Effects of *Gochujang* on Kidney Damage in HD‐Induced Obese Mice

To assess the effects of HD on kidney damage, both absolute and relative kidney weights were analyzed; additionally, serum BUN levels, renal KIM‐1 expression, and histological alterations in the kidney were investigated.

**FIGURE 4:**
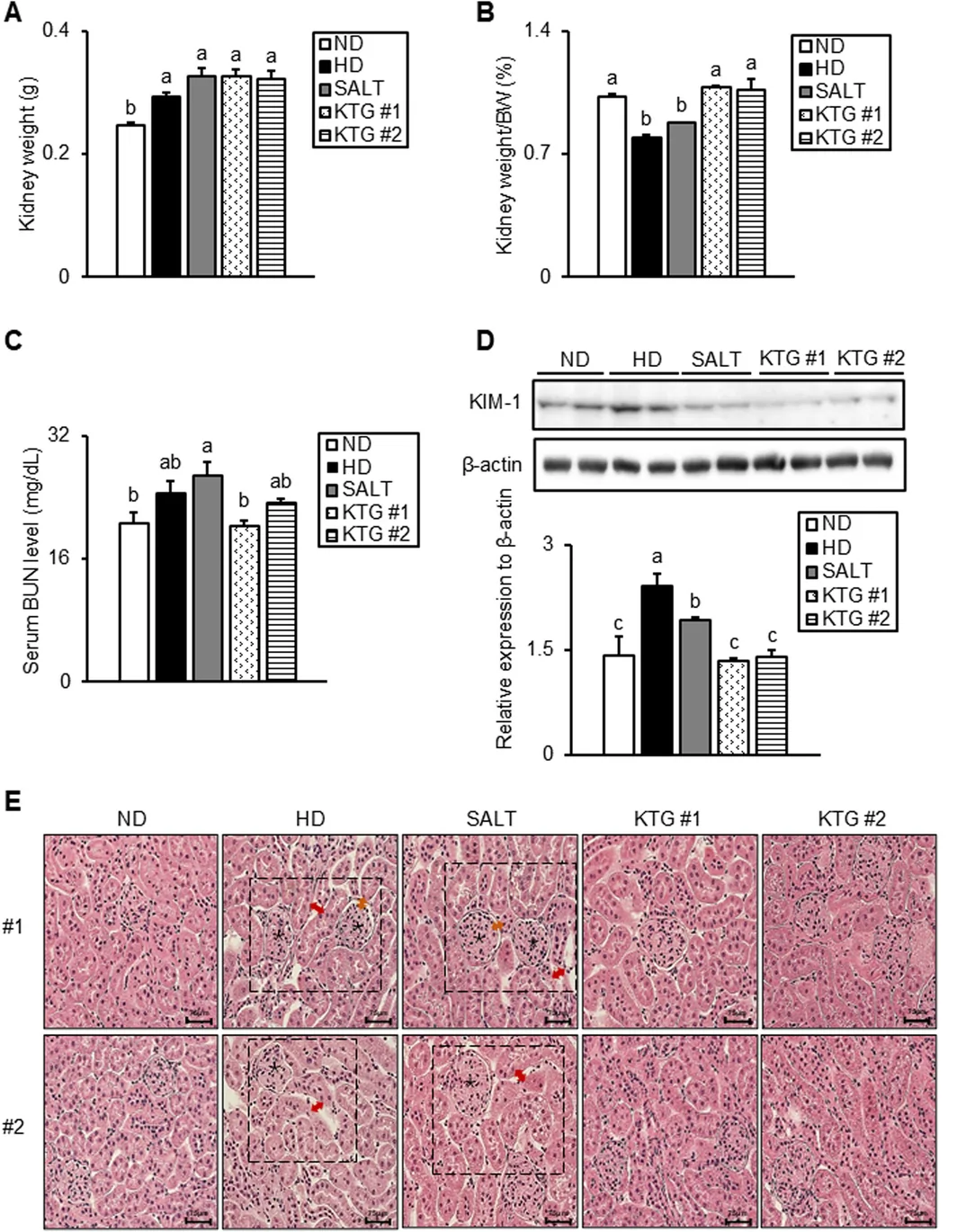
Effects of *Gochujang* on kidney damage in HD‐induced obese mice. (A) Kidney weight. (B) Kidney weight/body weight (BW) ratios in each experimental group. (C) Serum blood urea nitrogen (BUN) levels. (D) Kidney injury molecule‐1 (KIM‐1) protein levels were determined by western blotting in the kidney of each group. β‐actin was used as a loading control. Values are mean ± SEM. (a‐c) means with the different letters on the bar (a > b > c). (E) Histological analysis (40X) of kidney tissues by hematoxylin and eosin (H&E) staining. Scale bar = 75 μm. Two images per group were selected (#1 and #2). Black asterisk (*) indicates glomerular structural alterations. The red arrow indicates tubular dilation and interstitial expansion. The orange arrow indicates widened Bowman's space. ND, normal diet group; HD, high‐fat diet group; SALT, HD with 0.7% salt group; KTG #1, HD with 10% of KTG #1 *Gochujang* group; KTG #2, HD with 10% of KTG #2 *Gochujang* group.

Compared to ND group, HD, SALT, and all KTG groups exhibited a significant increase in absolute kidney weight (Figure 4A). However, the relative kidney weight was significantly lower in HD and SALT groups compared to ND group. In contrast, all KTG groups exhibited a significantly higher relative kidney weight than HD and SALT groups (Figure 4B). Compared to ND group, serum BUN levels were significantly increased in SALT group, while the increase in HD group was not statistically significant (Figure 4C). Conversely, all KTG groups had lower BUN levels compared to HD and SALT groups. Additionally, HD and SALT groups showed a significant increase in KIM‐1 levels compared to ND group (Figure 4D). Moreover, the KIM‐1 level of HD group was significantly higher than that of SALT group. All KTG groups had significantly lower KIM‐1 levels than the HD and SALT groups. Additionally, the renal α‐SMA expression was significantly decreased in HD and SALT groups compared to ND, whereas it was significantly increased in all KTG groups compared to HD and SALT (Figure S2). Lastly, compared to ND, both HD and SALT groups exhibited significant histopathological kidney damage characterized by glomerular structural alterations, tubular dilation, widening of Bowman's space, and expansion of the interstitial area (Figure 4E). In contrast, all KTG groups showed more organized glomerular and tubular structures, with reduced tubular dilation and less widening of Bowman's space, indicating an overall improvement in histopathological features.

Collectively, these findings indicate that *Gochujang* restores HD‐induced renal damage by improving serum BUN levels, kidney damage‐related protein expression, and histological impairments.

### 3.5 Effects of *Gochujang* on Renal Inflammation in HD‐Induced Obese Mice

To investigate *Gochujang*'s beneficial effects against HD‐induced renal inflammatory response, p‐IκB, p‐NF‐κB, and further downstream proteins associated with the inflammatory signaling pathway were probed.

**FIGURE 5:**
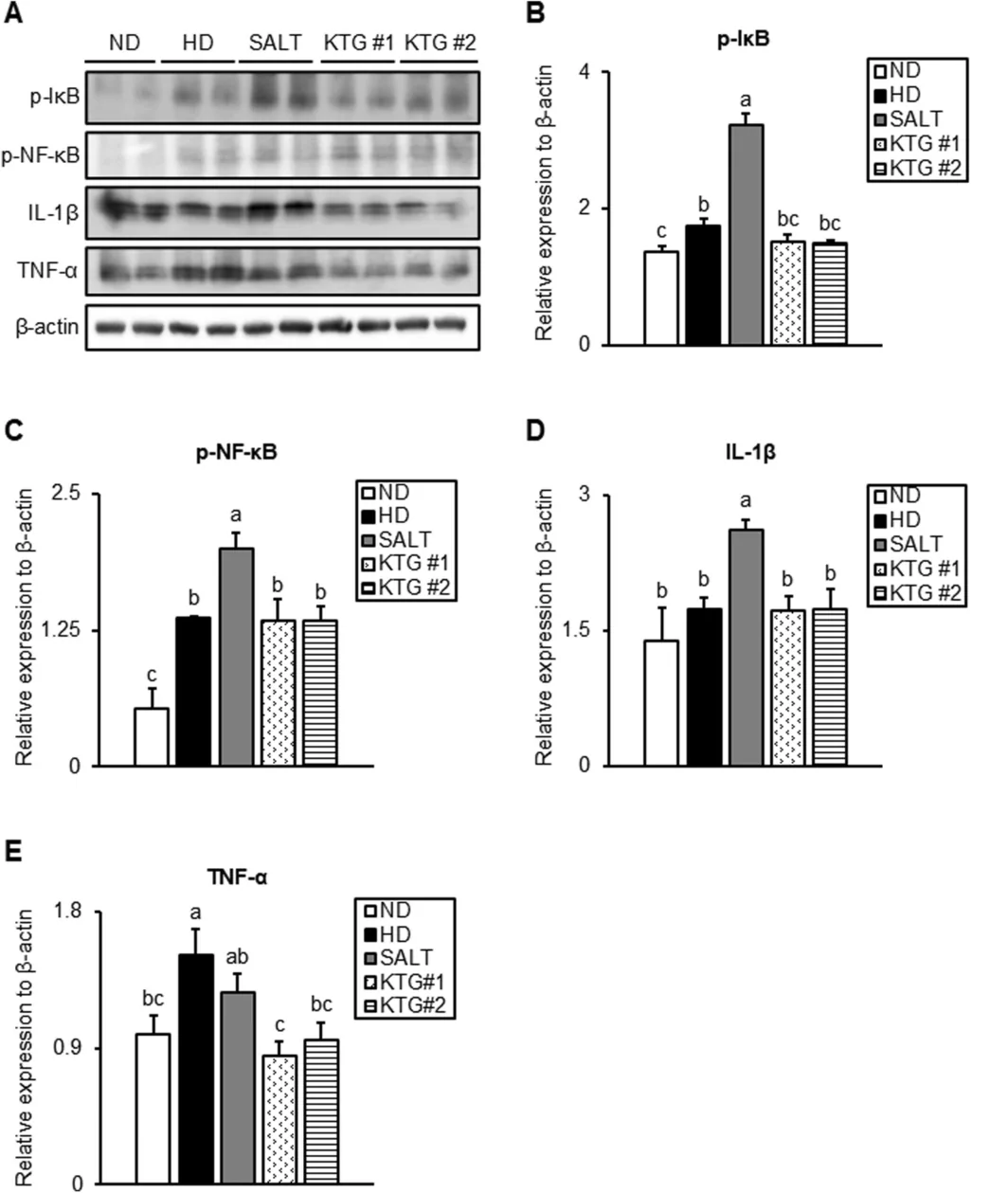
Effects of *Gochujang* on renal inflammation in HD‐induced obese mice. (A) Protein levels of p‐IκB, p‐NF‐κB, IL‐1β, and TNF‐α in kidney tissues were assessed by Western blotting. (B–E) Relative expression levels of each protein involved in the inflammatory response: (B) P‐IκB, (C) P‐NF‐κB, (D) IL‐1β, (E) TNF‐α. β‐actin was used as a loading control. Values are mean ± SEM. a‐c means with the different letters on the bar (a > b > c). ND, normal diet group; HD, high‐fat diet group; SALT, HD with 0.7% salt group; KTG #1, HD with 10% of KTG #1 *Gochujang* group; KTG #2, HD with 10% of KTG #2 *Gochujang* group; IL‐1β, Interleukin‐1 beta; IκB, inhibitor of κB; NF‐κB, nuclear factor kappa B; p‐, phospho‐; TNF‐α, tumor necrosis factor alpha.

Phosphorylation of IκB was significantly increased in both HD and SALT groups compared to the ND group, while all KTG groups showed a marked reduction of phospho‐IκB levels compared to the SALT group and were slightly lower than those in the HD group (Figure 5A,B). Phosphorylation of NF‐κB was also significantly elevated in both HD and SALT groups relative to the ND group, with a further increase observed in the SALT group compared with the HD group (Figure 5A,C). All KTG groups exhibited a significant decrease in phospho‐NF‐κB levels relative to the SALT group. Compared with the ND group, IL‐1β expression was significantly elevated in the SALT group, while no significant changes were observed in the other groups (Figure 5A,D). Furthermore, TNF‐α expression was significantly increased in the HD group compared with the ND group, whereas the SALT group showed a modest increase in TNF‐α levels (Figure 5A,E). All KTG groups showed lower TNF‐α expression than both HD and SALT groups. These findings suggest that while high salt intake accelerates the renal inflammatory response, *Gochujang* partially attenuates renal inflammation compared to salt alone intake, rather than inducing a comparable inflammatory response despite having the same salt content.

### 3.6 Effects of *Gochujang* on Gut Microbiota Imbalance in HD‐Induced Obese Mice

*Gochujang* improves HD‐induced hepatic inflammation by recovering gut microbiota imbalance (E.‐J. Lee et al. 2023). Thus, the impact of *Gochujang* on the abundance and diversity of gut microbiota was assessed.

Various α‐diversity indices, including ACE, Chao1, Shannon, Simpson, OTUs, and phylogenetic diversity, were analyzed. Compared to the ND group, the HD and SALT groups exhibited minimal or no significant changes in microbial abundance, while all KTG groups showed a significant increase in microbial species richness compared to the HD and SALT groups (Figure S3).

**FIGURE 6:**
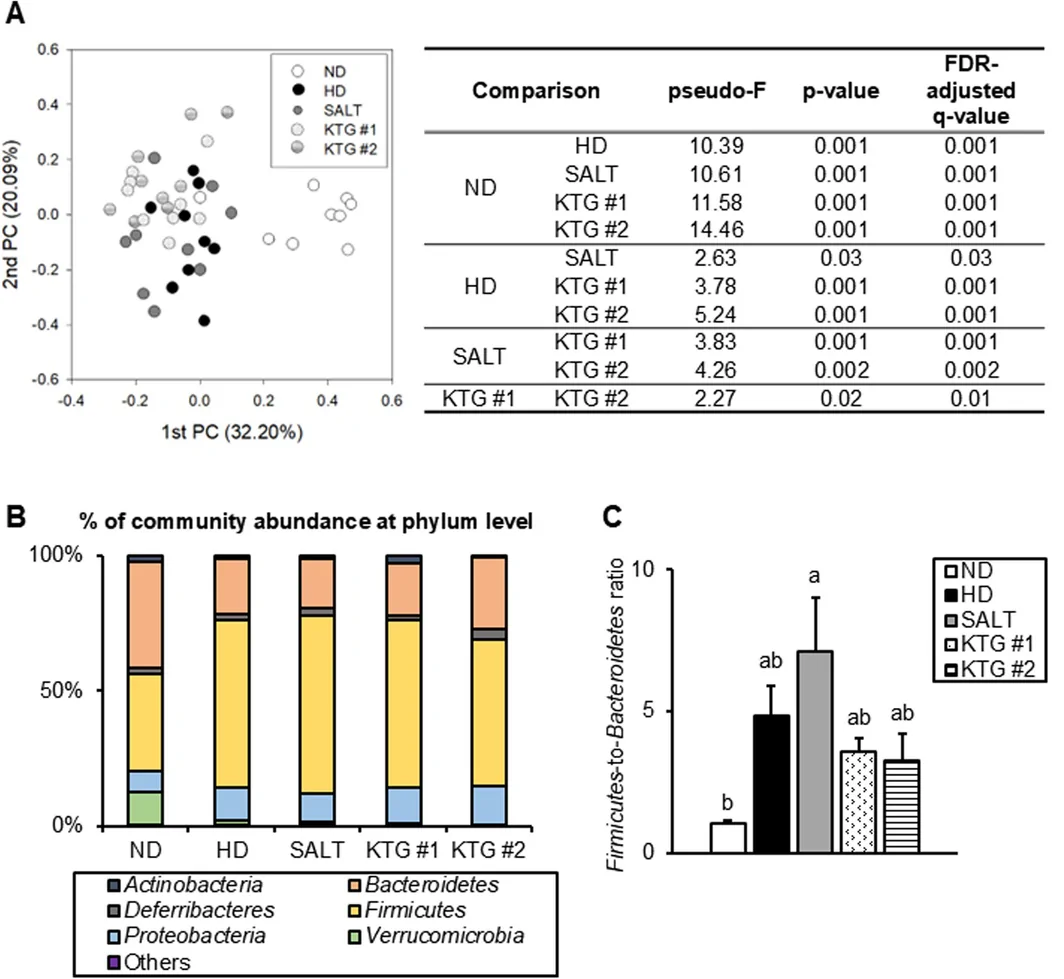
Effect of *Gochujang* on the population structure of gut microbiota at phylum level in HD‐induced mice. (A) Principal coordinate analysis (PCoA) plots based on the Bray–Curtis dissimilarity index. Pairwise PERMANOVA was performed with 999 permutations, and *q*‐values were adjusted for multiple comparisons using the false discovery rate (FDR) method. (B) Microbial community at the phylum level in mice. (C) *Firmicutes*‐to‐*Bacteroidetes* ratio in mice. Values are mean ± SEM. a‐b means with the different letters on the bar (*a* > b). ND, normal diet group; HD, high‐fat diet group; SALT, HD with 0.7% salt group; KTG #1, HD with 10% of KTG #1 *Gochujang* group; KTG #2, HD with 10% of KTG #2 *Gochujang* group.

Next, gut microbiota composition was analyzed at the phylum level. Additionally, to investigate differences in microbial community structure among the groups, β‐diversity was assessed using Bray–Curtis distance‐based principal coordinate analysis (PCoA) (Figure 6A). Bray–Curtis PCoA showed distinct microbial community distributions among the experimental groups. Firmicutes and Bacteroidetes were dominant in all groups (Figure 6B). In HD and SALT groups, the Firmicutes ratio was increased compared to the ND group, while the Bacteroidetes ratio was decreased. In contrast, all KTG groups showed a decrease in Firmicutes and an increase in Bacteroidetes compared to HD and SALT groups (Figure 6B). Therefore, the Firmicutes/Bacteroidetes ratio (F/B ratio) was significantly higher in the SALT group than in the ND group, whereas the HD group showed an increase without statistical significance (Figure 6C). All KTG groups showed a decreasing trend compared with both HD and SALT groups, although the differences were not statistically significant.

**FIGURE 7:**
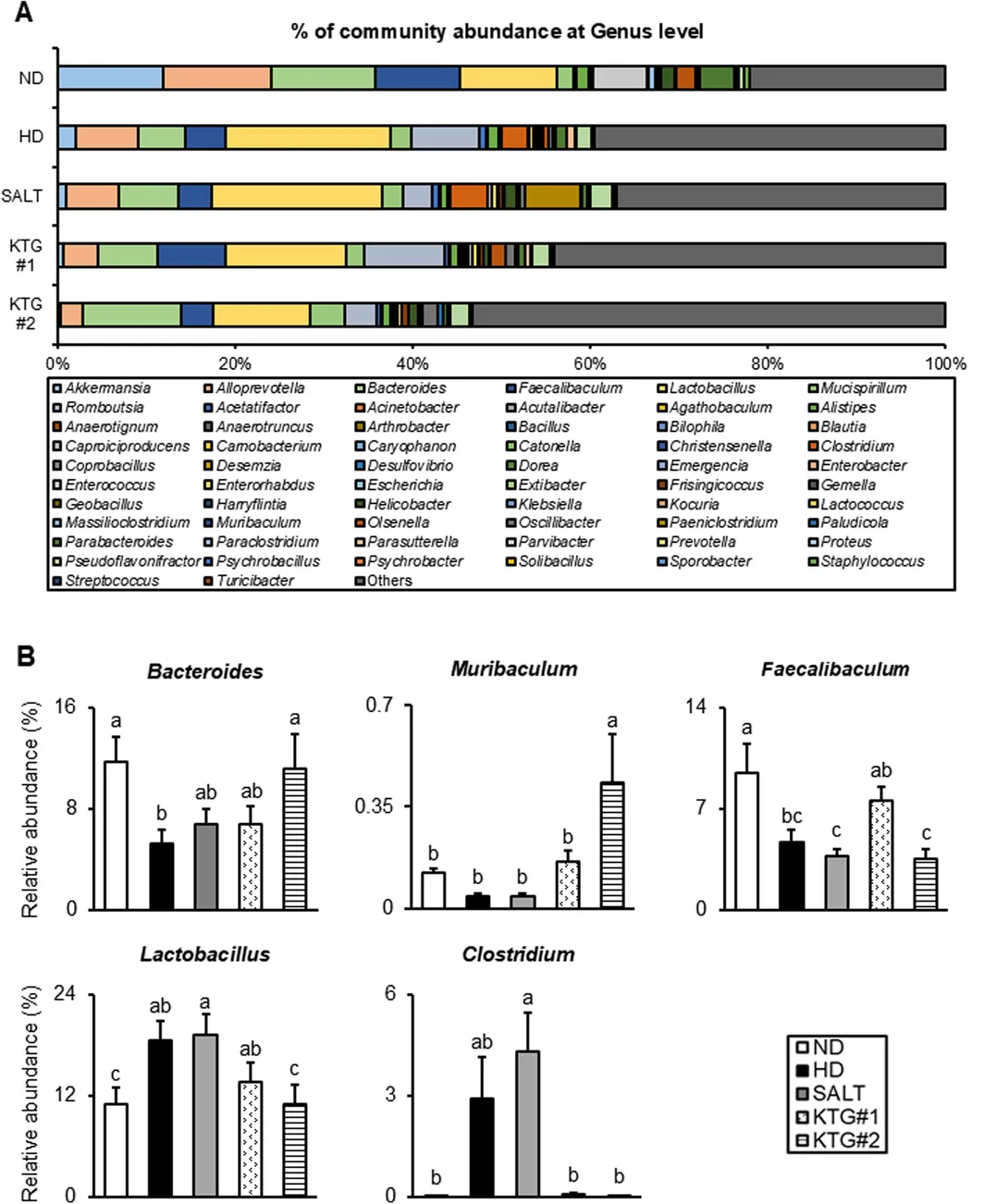
Effect of *Gochujang* on the population structure of gut microbiota at genus level in HD‐induced mice. (A) Microbial community at the genus level in mice. (B) The relative abundance of *Bacteroides*, *Muribaculum*, *Faecalibaculum*, *Lactobacillus*, and *Clostridium* at the genus level. Values are mean ± SEM. (a–c) means with the different letters on the bar (a > b > c). ND, normal diet group; HD, high‐fat diet group; SALT, HD with 0.7% salt group; KTG #1, HD with 10% of KTG #1 *Gochujang* group; KTG #2, HD with 10% of KTG #2 *Gochujang* group.

Based on the phylum‐level results, a detailed genus‐level analysis was also conducted, revealing clear changes in microbial communities across the groups (Figure 7A). Compared with the ND group, *Bacteroides* and *Faecalibaculum* were significantly decreased in the HD group, whereas *Muribaculum* showed no significant change. KTG groups generally increased these genera, with significant increases observed for *Bacteroides* and *Muribaculum* in KTG #2 and for *Faecalibaculum* in KTG #1 (Figure 7B, upper panel). Additionally, *Lactobacillus* and *Clostridium* were significantly increased in the HD and SALT groups compared to the ND group. These increases were attenuated in all KTG groups, with a more pronounced effect observed in the KTG #2 group (Figure 7B, lower panel).

In summary, these results suggest that *Gochujang* alters gut microbiota diversity and composition induced by HD.

### 3.7 Heatmap of Spearman Correlation Coefficients Between Key Gut Microbiota and Kidney Inflammation Traits

**FIGURE 8:**
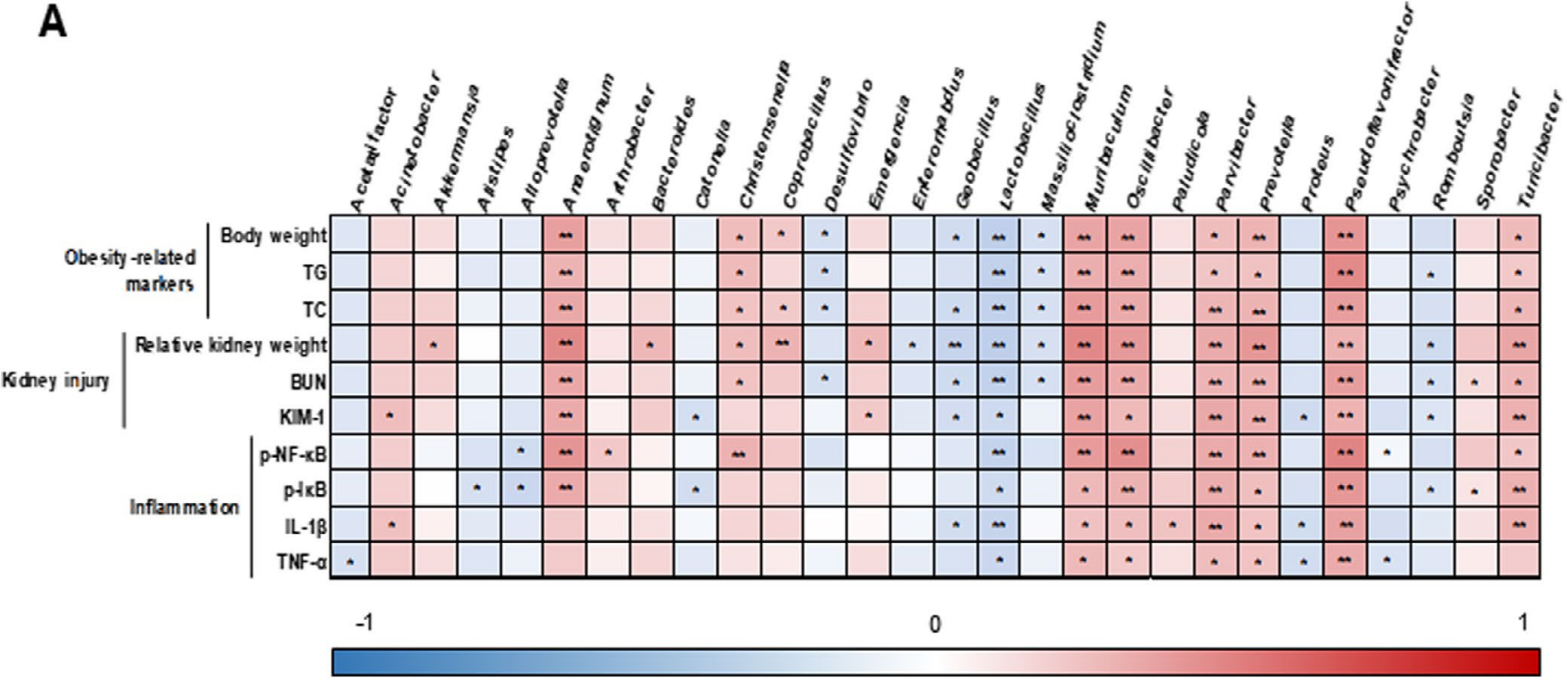
Heatmap of Spearman correlation coefficients between key gut microbiota and kidney inflammation traits. The intensity of the color represents the degree of association (blue, negative correlation; red, positive correlation). * (*p* < 0.05), ** (*p* < 0.01). ND, normal diet group; HD, high‐fat diet group; SALT, HD with 0.7% salt group; KTG #1, HD with 10% of KTG #1 *Gochujang* group; KTG #2, HD with 10% of KTG #2 *Gochujang* group.

To assess the correlation between key physiological markers of kidney damage and genus‐level alterations in the gut microbiota, Spearman's correlation coefficients were analyzed (Figure 8).

Several genera exhibited significant correlations with kidney injury and inflammatory markers. *Lactobacillus*, *Alistipes*, *Alloprevotella*, *Catonella*, *Geobacillus*, *Proteus*, and *Psychrobacter* were negatively correlated with parameters such as body weight, lipid levels, BUN, KIM‐1, and pro‐inflammatory cytokines (e.g., TNF‐α, IL‐1β, and p‐NF‐κB). Notably, *Lactobacillus* showed consistently negative associations across a wide range of metabolic and inflammatory markers. In contrast, genera including *Anaerotignum*, *Muribaculum*, *Oscillibacter*, *Parvibacter*, *Prevotella*, *Pseudoflavonifractor*, and *Turicibacter* displayed strong positive correlations with obesity‐related traits, kidney hypertrophy, and inflammatory indicators. Additionally, *Akkermansia* and *Bacteroides* were positively correlated with relative kidney weight, while *Acinetobacter* was associated with increased levels of KIM‐1 and IL‐1β. Other genera, such as *Christensenella*, *Coprobacillus*, and *Emergencia*, were also positively associated with multiple physiological parameters, such as relative kidney weight and body weight.

These correlations indicate an association between specific gut microbial genera and renal injury‐related indicators, suggesting that alterations of gut microbiota may be correlated with the renoprotective effects of *Gochujang*.

## 4 Discussion

*Gochujang* is well‐known for its multiple health‐beneficial effects, such as anti‐inflammatory effects (H. Lee et al. 2023); however, its renoprotective effects in HD‐induced kidney damage remain unexplored. Moreover, although there are concerns regarding its high salt content, the effects of *Gochujang* on kidney health have been discussed (Park et al. 2024). This study demonstrated that *Gochujang* effectively mitigated kidney damage by lowering serum BUN levels and protein expression levels associated with kidney damage and inflammation. In addition, *Gochujang* alters gut microbiota diversity and composition, which are significantly associated with improvements in kidney damage‐related parameters. Lastly, *Gochujang* exhibits those effects despite its high salt content.

Elevated serum BUN and kidney KIM‐1 levels lead to structural damage and inflammation in the kidney (Yu et al. 2022). In this study, *Gochujang* significantly reduces serum BUN and renal KIM‐1 levels in HD‐induced obese mice. Moreover, *Gochujang* strongly downregulates inflammatory response‐associated protein levels in both HK‐2 cells and the kidneys of HD‐induced obese mice. It is speculated that diverse bioactive compounds in *Gochujang* might be responsible for these improvements (Guerreiro et al. 2022). For example, capsaicin and genistein, the major bioactive compounds in *Gochujang*, themselves strongly reduce inflammation and oxidative stress in the kidney tissue (Peng et al. 2022; Ran et al. 2022). However, the exact compounds of *Gochujang* and the precise mechanisms for kidney health have not been fully investigated yet, emphasizing the need for future investigations. Moreover, histological analysis demonstrated that *Gochujang* attenuated renal structural alterations, including tubular dilation and interstitial expansion, in HD‐induced obese mice. These observations support the potential role of *Gochujang* in mitigating renal injury associated with metabolic stress. Therefore, further studies should also explore the effects of *Gochujang* on renal oxidative stress and lipotoxicity to elucidate the diverse mechanisms underlying its renoprotective effects. Unexpectedly, the present study found that *Gochujang* increased α‐SMA expression despite improvements in other renal injury‐related markers. Although α‐SMA is commonly recognized as a marker for fibrosis, it may also play a critical role in the recovery of renal tissue by activation of the Wnt/β‐catenin signaling pathway (Fujigaki et al. 2005; Gonlusen et al. 2001; Ran et al. 2022; Takano et al. 2007; Zhou et al. 2019). However, the exact role of *Gochujang* in α‐SMA regulation remains unresolved and requires further investigation by analyzing additional fibrosis‐ and repair‐related markers.

The effects of *Gochujang* on the recovery from gut microbiota dysbiosis and its association with improvements in metabolic disease have been reported (Kim et al. 2023; E.‐J. Lee, song, et al. 2023; Malesza et al. 2021). For example, *Gochujang* improves HD‐induced hepatic inflammation by restoring gut microbiota imbalance. Consistent with previous findings, the current study also found that *Gochujang* significantly alters gut microbiota diversity and composition. Furthermore, *Gochujang* increases the abundance of *Bacteroides*, *Muribaculum*, and *Faecalibaculum*, but decreases *Lactobacillus* and *Clostridium*. Importantly, this study found that *Gochujang*‐induced changes in the gut microbiota are strongly associated with indicators of kidney damage, suggesting a possible relationship between gut microbiota modulation and the renoprotective effects of *Gochujang*. *Bacteroidetes* play a role in inhibiting inflammatory cytokines and in the production of short‐chain fatty acids (SCFAs) (Mao et al. 2023). Additionally, *Muribaculum* modulates inflammation, enhances gut barrier function, and promotes SCFA production (Mun et al. 2019). Given the important role of the gut microbiota in SCFA production, future studies should investigate whether *Gochujang* alters microbial SCFA production and whether these metabolites causally contribute to the gut–kidney interactions associated with its renoprotective effects.

The high salt contents in KTFFs have been problematically discussed; however, it has been continuously reported that salt in KTFFs and table salt yield different health outcomes (the Korean paradox) (E.‐J. Lee, song, et al. 2023; Mun et al. 2019; Park et al. 2024; Woo et al. 2023). Lee et al. reported that *Gochujang* ameliorates obesity‐induced inflammation and hepatic damage, regardless of its high salt content (E.‐J. Lee et al. 2023). Mun et al. also demonstrated that *Doenjang*, another representative KTFF, does not cause hypertension, while the same amount of table salt significantly induces hypertension (Mun et al. 2019). Furthermore, Woo et al. showed that *Doenjang* exerts anti‐obesity and anti‐hypertensive effects in HD‐induced obese mice by modulating the renin‐angiotensin system (RAS), despite its high salt content (Woo et al. 2023). Consistent with previous findings, the current study observed that table salt significantly causes kidney damage, more than HD, whereas *Gochujang* dramatically mitigates HD‐induced renal damage, despite its high salt concentration. Recently, Park et al. also reported that *Gochujang* improves blood pressure by regulating the RAS and sodium homeostasis, despite its high salt content, thereby exerting antihypertensive effects (Park et al. 2024).

There are a few limitations in this study. First, although the overall renoprotective effects of *Gochujang* were evaluated by integrating serum BUN levels, renal KIM‐1 expression, histological observations, and inflammatory markers, the present study assessed only a single serum marker of kidney function. Since serum BUN levels can be affected by factors other than renal function (e.g., dietary protein intake), additional serum markers of kidney function, including creatinine, should be evaluated in future studies to provide a more comprehensive assessment of *Gochujang*'s effects on kidney injury‐related changes. Second, although the current study evaluated the TPC, TFC, and antioxidant activities of *Gochujang*, its detailed bioactive compounds were not fully characterized. Therefore, further studies are needed to identify specific bioactive compounds (e.g., capsaicin) and clarify their individual contributions to the renoprotective effects of *Gochujang*. Lastly, although alterations in the gut microbiota were significantly associated with renal injury and inflammatory indicators, the current study did not establish causality, requiring future studies to determine whether the gut microbiota mediates the renoprotective effects of *Gochujang*.

In conclusion, despite its high salt content, *Gochujang* attenuated obesity‐induced kidney injury‐related changes. In the future, clinical studies are required to confirm these effects on obesity‐related kidney injury.

## 5 Conclusion

This study demonstrated that *Gochujang* markedly inhibits lipid accumulation and inflammatory responses in both in vitro and in vivo models. Additionally, *Gochujang* modulates the gut microbiota by lowering the *Firmicutes*‐to‐*Bacteroidetes* ratio. Moreover, those alterations in gut microbiota diversity and composition are markedly correlated with the improvements in renal injury indicators. Importantly, despite their distinct microbial compositions, both *Gochujang* consistently attenuated obesity‐induced inflammatory kidney damage, accompanied by comparable alterations in the gut microbiota. Furthermore, despite its high salt content, *Gochujang* still exhibits stronger kidney‐protective effects than table salt, supporting the “Korean paradox”. These findings support the potential of *Gochujang* as a functional food to alleviate obesity‐related kidney injury, although further mechanistic and clinical studies are required.

## Author Contributions

**So‐Min Oh:** conceptualization, investigation, methodology, data curation, visualization, writing – original draft, formal analysis. **Un‐Seong Na:** investigation, data curation, formal analysis, visualization, writing – review and editing. **Do‐Youn Jeong:** resources, project administration, funding acquisition. **Hayoung Woo:** conceptualization, methodology, validation, writing – review and editing. **Anna Han:** supervision, data curation, visualization, writing – review and editing.

## Funding

This research was supported by the National Research Foundation of Korea (NRF) grant funded by the Korean government (Ministry of Science and ICT) (No. 2022R1F1A1064002). This research was funded by the “Functional identification of Korean traditional soybean products (safety monitoring) project” under the Ministry of Agriculture, Food and Rural Affairs, and partly by Korea Agro‐Fisheries and Food Trade Corporation in 2026.

Declaration of Generative AI Use: The graphical abstract was created using FigureLabs (https://www.figurelabs.ai/), an AI‐assisted scientific illustration tool. The authors reviewed and edited the final graphical abstract and take full responsibility for its accuracy and content.

## Ethics Statement

The animal study protocol was approved by the Institutional Animal Care and Use Committee of Jeonbuk National University (JBNU 2021‐0112).

## Conflicts of Interest

The authors declare no conflicts of interest.

## Supporting information


**Figure S1:** Nutritional composition of *Gochujang* and experimental diets.
(A) The nutritional composition of *Gochujang*. (B) The composition of the experimental diet. ND, normal diet group; HD, high‐fat diet group; SALT, HD with 0.7% salt group; KTG #1, HD with 10% of KTG #1 *Gochujang* group; KTG #2, HD with 10% of KTG #2 *Gochujang* group.

**Figure S2:** Effects of *Gochujang* on α‐SMA expression in HD‐induced obese mice.
(A) Alpha‐smooth muscle (α‐SMA) protein levels were determined by western blotting in the kidney of each group. β‐actin was used as a loading control. Values are mean ± SEM. a‐c means with the different letters on the bar (a > b > c). ND, normal diet group; HD, high‐fat diet group; SALT, HD with 0.7% salt group; KTG #1, HD with 10% of KTG #1 *Gochujang* group; KTG #2, HD with 10% of KTG #2 *Gochujang* group.

**Figure S3:** Relative abundance of the gut microbial community.
(A) Relative abundance of the gut microbial community. Values are mean ± SEM. a‐d means with the different letters on the bar (a > b > c > d). ND, normal diet group; HD, high‐fat diet group; SALT, HD with 0.7% salt group; KTG #1, HD with 10% of KTG #1 *Gochujang* group; KTG #2, HD with 10% of KTG #2 *Gochujang* group.

## Data Availability

The data that support the findings of this study are available from the corresponding author upon reasonable request.
